# Pre-Vaccination Nasopharyngeal Pneumococcal Carriage in a Nigerian Population: Epidemiology and Population Biology

**DOI:** 10.1371/journal.pone.0030548

**Published:** 2012-01-24

**Authors:** Ifedayo M. O. Adetifa, Martin Antonio, Christy A. N. Okoromah, Chinelo Ebruke, Victor Inem, David Nsekpong, Abdoulie Bojang, Richard A. Adegbola

**Affiliations:** 1 Medical Research Council (MRC) Unit, The Gambia, Fajara, The Gambia; 2 Department of Paediatrics and Child Health, College of Medicine, University of Lagos, Idi-Araba, Lagos, Nigeria; 3 Department of Community Health and Primary Care, College of Medicine, University of Lagos, Idi-Araba, Lagos, Nigeria; National Institute of Child Health and Human Development, United States of America

## Abstract

**Background:**

Introduction of pneumococcal vaccines in Nigeria is a priority as part of the Accelerated Vaccine Introduction Initiative (AVI) of the Global Alliance for Vaccines and Immunisation (GAVI). However, country data on the burden of pneumococcal disease (IPD) is limited and coverage by available conjugate vaccines is unknown. This study was carried out to describe the pre vaccination epidemiology and population biology of pneumococcal carriage in Nigeria.

**Methods:**

This was a cross sectional survey. Nasopharyngeal swabs (NPS) were obtained from a population sample in 14 contiguous peri-urban Nigerian communities. Data on demographic characteristics and risk factor for carriage were obtained from all study participants. Pneumococci isolated from NPS were characterised by serotyping, antimicrobial susceptibility and Multi Locus Sequencing Typing (MLST).

**Results:**

The prevalence of pneumococcal carriage was 52.5%. Carriage was higher in children compared to adults (67.4% vs. 26%), highest (≈90%) in infants aged <9 months and reduced significantly with increasing age (P<0.001). Serotypes 19F (18.6%) and 6A (14.4%) were most predominant. Potential vaccine coverage was 43.8%, 45.0% and 62% for PCV-7, PCV-10 and PCV-13 respectively. There were 16 novel alleles, 72 different sequence types (STs) from the isolates and 3 Sequence Types (280, 310 and 5543) were associated with isolates of more than one serotype indicative of serotype switching. Antimicrobial resistance was high for cotrimoxazole (93%) and tetracycline (84%), a third of isolates had intermediate resistance to penicillin. Young age was the only risk factor significantly associated with carriage.

**Conclusions:**

Pneumococcal carriage and serotype diversity is highly prevalent in Nigeria especially in infants. Based on the coverage of serotypes in this study, PCV-13 is the obvious choice to reduce disease burden and prevalence of drug resistant pneumococci. However, its use will require careful monitoring. Our findings provide sound baseline data for impact assessment following vaccine introduction in Nigeria.

## Introduction

Pneumonia is one of the leading causes of mortality in children <5 years old. It is responsible for 1.6 million of 8.8 million annual deaths in this age group [1] with 50% occurring in sub-Saharan Africa [2]. *Streptococcus pneumoniae* (pneumococcus), accounts for 30–50% of pneumonia-related deaths [3], [4], [5]. Pneumococcus is also a major cause of acute otitis media (AOM), bacteraemia, meningitis and sinusitis particularly in developing countries [5].

There are over 91 serotypes of pneumococci and worldwide, 6–11 serotypes are responsible for ≥70% of invasive disease (IPD) in children <5 years old [6]. The distribution of pneumococcal capsular serotypes found in nasopharyngeal (NP) carriage, and IPD vary by age, geography and socioeconomic status [7], [8], [9]. Pneumococcus is also part of the normal microbial flora in the nasopharynx, its main reservoir. Although harmless and asymptomatic by itself, carriage is a precondition for invasive and non-invasive disease and is associated with the incidence of AOM, bacteraemia and pneumonia [10], [11]. Pneumococcal carriage is highly prevalent in developing countries particularly among children aged <5 years. Carriage in <5 years age group varies from 44–90% in Papua New Guinea, Fiji, Indonesia, Venezuela and The Gambia [12], [13], [14], [15].

Since vaccination is the most effective public health tool to prevent IPD, pneumococcal conjugate vaccines (PCVs) containing 7, 10 and 13 serotypes have been developed although PCV-7 is being replaced by PCV-10 and -13 because it contains limited serotypes. Unlike the 23 serotype-containing polysaccharide vaccine, PCVs are more immunogenic in young children, induce immunological memory and provide serotype-specific protection against pneumococcal carriage. Although they contain serotypes most associated with disease and antimicrobial resistance worldwide, variation of serotypes geographically and antimicrobial resistant strains prevent universal protection. In addition, replacement in carriage and invasive disease by non-vaccine containing and drug resistant serotypes of pneumococci occurs with use of PCVs [16].

In many countries needing PCV the most, data on the burden of pneumococcal carriage and disease; as well as the prevalent serotypes and coverage by PCVs is limited or unknown. Nigeria is one of the 10 countries where two-thirds of all deaths due to pneumonia in children <5 years occur [17], [18]. It is also one of the 15 countries with the highest estimated number of cases of clinical pneumonia [3]. Over 30 years ago, pneumococci serotypes 1, 2, 3 and 5 were the serotypes associated with IPD in children and adults in North East, Nigeria. [19] A decade later, Silverman *et. al.* [20] found 51% of isolates from children with severe pneumonia were pneumococci. More recently, a small study in Eastern Nigeria found 69% pneumococci carriage rates in children <5years attending routine paediatric clinics [21]. And in a hospital based surveillance of invasive bacterial infections, pneumococcus was implicated in the majority of pneumonia, meningitis and bacteraemia in the children studied [22]. Existing data on pneumococcal carriage and disease in Nigeria have limitations including small sample sizes, poor yield of pneumococci and/or that very few isolates are serotyped and results from earlier studies are out of date. Consequently, the current prevalence of serotypes in carriage and disease is largely unknown in Nigeria. Baseline pneumococcal carriage and invasive rates data are crucial for assessing the impact of vaccination and for monitoring serotype changes and antimicrobial resistance patterns.

We investigated pneumococcal carriage in a Nigerian population to determine carriage rates, prevalent serotypes; theoretical coverage of pneumococcal conjugate vaccines and molecular epidemiology of pneumococci in this population.

## Methods

### Study area and population

The Lagos University Teaching Hospital Primary Health Care and Rural Medicine Centre (LUTH PHC), Pakoto village is approximately 70 kilometres from Lagos, Nigeria. This health centre serves all communities within a 10 km radius covering two local government areas (LGAs) and it was the base for this study. Pakoto is one of 250 communities in Ifo LGA, Ogun State, Nigeria. The total population is 350,000 (2006 National Population Census, Nigeria). Majority of the inhabitants are Yoruba.

### Design and Sample Size considerations

This was a cross sectional survey. The target sample was a range of 384–518 children aged <5 years required to estimate the prevalence of pneumococcal carriage with a precision of 5% (with 95% confidence) and 80–90% power. Since prevalence of carriage in the whole community has an impact on prevalence of carriage in children aged <5 years and vice versa, a sample including all age groups was preferred. To obtain a representative sample in an area with unknown age distribution, all consenting children aged <5 years; 1 in 3 persons aged 5–49 years and all those aged >50 years up to 1000 subjects were swabbed. Even if the proportion of children aged <5 years in the study communities was less than 25%, the sampling was designed to give an expected ratio of subjects of 25∶21∶11 (i.e. at least 440 <5 years). Communities selected for the study were within a 5 km radius of the health centre.

### Eligibility

All consenting adults and children ordinarily resident in the study area were eligible for enrolment.

### Ethical Considerations

The Ethics Committees of the Lagos University Teaching Hospital, Lagos, Nigeria, the London School of Hygiene and Tropical Medicine, UK approved this study and acknowledgement received from the Gambian Government and MRC joint ethics committee. Parents/guardians of minors and every adult participant gave written informed consent before participation in study. Those 15–17 years old gave assent was in addition to written consent given by parents/guardians.

### Data and Sample collection and identification of pneumococcus

A questionnaire was administered to each subject (adolescent with their parents/guardians and adults) or to their parent or guardian (for minors) by a trained field worker to obtain demographic, personal, clinical, general and age-specific risk factors data. The risk factors for which data was obtained via questionnaire were household smoking, number of <5-year old residents in household, household cooking fuel, breastfeeding in children aged <6 months, antibiotic use, gender, age, ethnicity, location, overcrowding, and socioeconomic status.

All procedures (swabbing, storage and culture) were carried out using standard methods recommended by the World Health Organization (WHO) [23]. Briefly, samples were obtained with a deep nasopharyngeal swab (NPS) using calcium alginate swab tips on a flexible aluminium shaft (Fisher brand®, Fisher Scientific, Pittsburgh, USA). NPS were placed inside Skim milk-tryptone-glucose-glycerin (STGG) transport media [24] and transported within 8 hours of collection for storage at −30°C before transportation in dry ice to the MRC. Samples were cultured at the MRC Microbiology Laboratory on gentamicin 5% sheep blood agar incubated at 37°C in 5% CO_2_. Samples of all typical *S. pneumoniae* colonies obtained by sweep method from the plates were subjected to pneumococcal identification methods of α-haemolysis, colony morphology and ethylhydrocupreine hydrochloride (optochin). Bile solubility testing was applied to isolates with intermediate optochin sensitivity. Serotyping was done by latex agglutination using serotype specific antisera (Statens Serum Institute, Denmark). Confirmation of equivocal isolates was by molecular serotyping using multiplex PCR [25]. As *S. pneumoniae* serotype 6A cross-reacts serologically with serotype 6C, we tested all serotype 6A isolates by multiplex PCR to differentiate serotype 6A from 6C as previously described [26]. Isolates that were non-typeable by these methods were listed as non-typeable pneumococcus.

Randomly selected isolates (201) had to microbial susceptibility tests to Penicillin G (PG), Cefotaxime (CT), Tetracycline (TC), Trimethoprim/sulfamethoxazole (TS), Erythromycin (E) and Chloramphenicol (CL) using E-tests (AB Biodisk, Solna, Sweden). Minimum inhibitory concentrations (MICs) with resistance and sensitivity according to the interpretive criteria provided in the product insert were recorded. Resistant Isolates were those with MICs above the threshold for sensitivity.

The MRC Unit, submits to the external quality assurance programme of the United Kingdom National External Quality Assessment Service [27] and is a World Health Organization (WHO) regional reference laboratory for invasive bacterial pathogens.

### Multi locus sequencing typing (MLST)

MLST was performed as previously described [28]. Sequence types (STs) were analyzed for relatedness using the eBURST v3 program. [29] Cluster analysis of allelic profiles was performed using a categorical coefficient and a graphic method called a minimum spanning tree with Bionumerics software © (version 4.0; Applied Maths, Sint-Martens-Latem, Belgium).

### Data Management and Statistical Analysis

All data were double entered into an Access relational database. Statistical analyses were performed using Stata 11.1 (Stata Corp LP, College Station, Texas). The main outcome variable was prevalence of pneumococcal carriage. The secondary outcomes were genetic structure of and serotypes found in carriage, their antibiotic susceptibility and risk factors (both general and specific) associated with carriage. Analysis of carriage was done with ‘person’ as unit of analysis while analysis of serotypes and antibiotic resistance was done with ‘isolate’ as unit of analysis.

The exposures considered were household smoking, number of <5-year old residents in household, household cooking fuel, breastfeeding in children aged <6 months, antibiotic use, gender, age, ethnicity, location, overcrowding, and socioeconomic status. Socioeconomic status was assessed using a modification of the socio-economic index score validated for Nigeria [30]. Multivariate analyses were carried out using logistic regression. Analysis of each exposure/risk factor was adjusted for sex and age and adjusted odds ratios for the presence of carriage were obtained separately for each risk factor.

Random effects logistic regression models were used to adjust for the effect of clustering on outcomes by location.

## Results

### General characteristics of study population

Out of 1025 consenting participants, 98.0% (1005) had useable NPS that were cultured. Their median age was 4.4 (interquartile range [IQR] 1.3–30) years. They were predominantly female (652, 63.8%) and Yoruba (952, 92.8%). As planned, there were more children (654, 63.8%) than adults were. A history of antibiotic use in sampled children was obtained in 100 (15.6%) of 641 children and from 76 out of 361 (21.1%) adults. Overall, 176 (17.5%) of total study population had taken a course of antibiotics at least 1 month prior to sampling. Antibiotics were obtained mostly by self-prescription and purchased from drug stores (66.7% [95% CI 54.8–77.1%], 50 of 75).

### Prevalence of carriage

**Figure 1:**
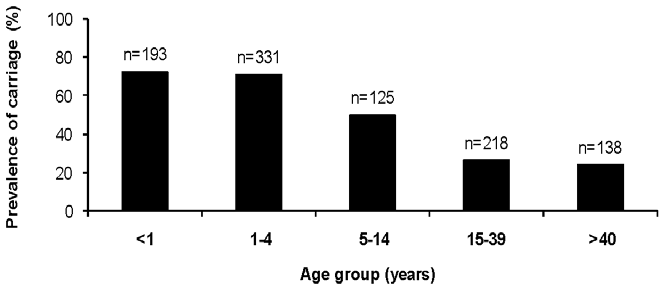
Prevalence of pneumococcal carriage by age group in a peri-urban Nigerian Community.

Pneumococcus was identified in swabs from 528 of 1005 subjects. The prevalence of carriage was 52.5% (95% Confidence Interval [CI]: 49.4–55.7%). As seen in figure 1, the prevalence of carriage reduced significantly with increasing age (χ^2^ = 165.7, p<0.0001 for trend).

**Figure 2:**
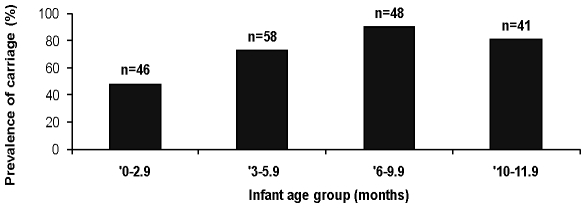
Prevalence of nasopharyngeal carriage of pneumococcus by infant age category in a peri urban Nigerian Community.

Pneumococcal carriage was higher in children (those <18 years), 434 of 644 (67.4% [95% CI 63.6–71%]) compared to adults, 94 of 361 (26% [95% 21.6–30.9%] and this difference was significant (p<0.001). Carriage was highest (244 of 328) in children aged <2 years i.e. 74.4% (69.3%–79.0%). As shown in figure 2, pneumococcal carriage steadily increased from 47.8% very early in infancy (<2.9 months) to peak values of 89.6% between 6–9 months and this trend was statistically significant (χ^2^ = 15.3, p = 0.0001). There was a higher prevalence of pneumococcal carriage in males compared to females (60.3% vs. 48.0%, p<0.001). However, when subjects were stratified into adults or children, this difference was no longer statistically significant. Pneumococcal carriage was also unrelated to ethnicity (52% in Yoruba vs. 53% in other ethnic groups, p = 0.9).

### Serotypes, clones in carriage and theoretical vaccine coverage

Forty-two (42) serotypes of pneumococcus were identified, while 17 isolates (3.2%) were non-typeable (NT). The distribution of serotypes also varied by age group with almost 80% of all serotypes carried by children <5 years diminishing to ≈50% in the older age groups as shown in Table 1. The top 10 serotypes found and the age groups related differences in the ranking of prevalence serotypes are shown in Table 1. There were 3–5 vaccine serotypes within the top 10 ranked serotypes in all age categories. The fewest number (3) was found in the 5–14 years category. About 9.3% carried multiple pneumococcal serotypes (49 of 528). Carriage of multiple pneumococci was associated mainly with serotypes 19F (2.7%) and 6A (2.3%) while serotypes 6B and 23F together were associated with carriage of multiple serotypes in 2.5% of subjects.

**Table 1:** Pneumococcal Carriage and approximate vaccine coverage by age.

	All	<5years	5–14 years	>15–39years	≥40
**Numbers with carriage (n)**	528	375	63	57	33
**Carriage (%)**	52.5	71.0	11.9	10.8	6.3
**Number of different serotypes seen**	42	33	21	21	19
**Top 10 Serotypes**					
**1**	19F (94)	19F (71)	19F (10)	19F (11)	11 (3)
**2**	6A (76)	6A (65)	11 (7)	6A (7)	23F (3)
**3**	6B (72)	6B (60)	NT (6)	23F (6)	3 (3)
**4**	23F (37)	23F (27)	18C (5)	11 (4)	6B (3)
**5**	11 (27)	15B (14)	6B (5)	18C (4)	12 (2)
**6**	15B (21)	11 (13)	3 (4)	6B (4)	13 (2)
**7**	3 (19)	14 (13)	6A (4)	15B (3)	17 (2)
**8**	NT (17)	19A (10)	15B (3)	3 (2)	19F (2)
**9**	18C (15)	3 (10)	20 (3)	4 (2)	7C (2)
**10**	9V (15)	9V (10)	21 (3)	7F (2)	9V (2)
**Total isolates represented by top 10 serotypes**	74.4%	78.1%	79.4%	78.9%	72.7%
**PCV-7 Serotypes**	231 (43.8%)	173 (46.1%)	19 (30.2%)	28 (49.1%)	11 (33.3%)
**PCV-10 Serotypes**	235 (44.5%)	174 (46.4%)	20 (31.8%)	30 (52.6%)	11 (33.3%)
**PCV-13 Serotypes**	325 (61.6%)	264 (70.4%)	27 (42.9%)	38 (66.7%)	14 (42.4%)

**PCV**-pneumococcal conjugate vaccine; **NT**-Non Typeable; **PCV7 serotypes**: 4, 6B, 9V, 14, 18C, 19F, 23F; **PCV10 serotypes**: PCV7+ 1, 5, 7F; **PCV13 serotypes**: PCV10+ 3, 6A, 19A.

Serotypes9V, 14, 18C, 3 and 15C accounted for 2–4% of overall pneumococcal isolates, while other serotypes not previously mentioned made up less than 2% of all serotypes found.

The theoretical coverage for the earliest available conjugate vaccine-PCV7, was poor for all age categories ranging from 30.2%–49.1% and there was very little difference between the coverage offered by the newer PCV10 when compared to PCV7 (Table 1). In children aged <2 years, coverage was identical for PCV7 and PCV10, 34.8% and was 61.7% for PCV13. For all PCVS, coverage was highest for the <5 years and ≥15–39 years age group as seen in Table 1.

### Population snapshot of pneumococcal isolates by MLST: discovery of novel alleles

**Figure 3:**
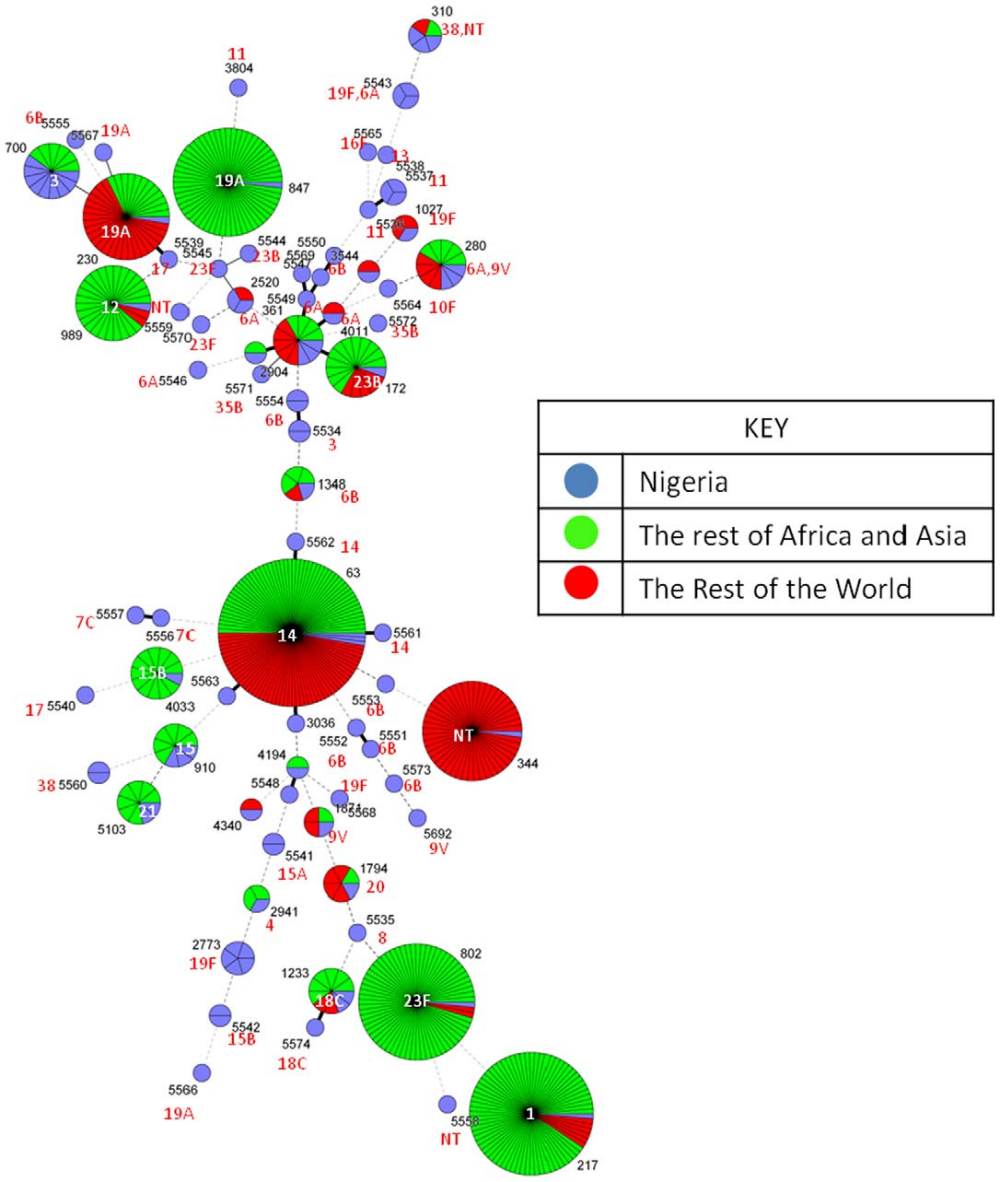
Clustering of STs by use of the minimum spanning tree. Each circle represents an ST. The area of each circle corresponds to the number of isolates. Thick, short, solid lines connect single locus variants; thin longer solid lines connect double locus variants and broken lines connect three or more locus variants. Texts in red and inside the circles are the serotypes.

MLST was performed on 99 pneumococcal isolates representing 29 different serotypes selected randomly from the 528 pneumococcal isolates. We found 16 novel alleles [(*aroE* (172, 176, 177), *gdh* (206, 260, 261, 262); *gki* (260, 261); *recP* (154), *spi* (239, 244), *xpt* (346, 354, 355), *ddl 406*)] and with 72 (72.7%) different sequence types (ST) as shown in table S1. e-burst analysis using the stringent 6/7 identical loci definition grouped these STs into 9 clonal complexes of closely related strains and 45 singletons (diagram not shown). The higher number of singletons (83.3%) identified in the study *i.e.* STs with no close relatives reflects a highly diverse pneumococcal population structure in Nigeria, which is different from those STs in the global MLST database (figure 3). Three STs (280, 310 and 5543) were found to be associated with isolates of more than one serotype indicative of serotype switching (see Table S1).

### Antimicrobial susceptibility

A random selection of 201 pneumococcal isolates was tested for susceptibility to the following antibiotics; Erythromycin (E), Tetracycline (TC), Cefotaxime (CT), Trimethoprim-Sulphamethoxazole (TS), Penicillin G (PG) and Chloramphenicol (C). Isolates were only fully susceptible to erythromycin and 1 (0.5%) isolate had high-level resistance to CT otherwise others were fully susceptible (see table 2). Of all the isolates resistant to tetracycline, 75.2% (151) and 9.0% (18) had high level and intermediate resistance respectively. The highest level of resistance overall was seen with TS with high level and intermediate resistance in 110 (54.7%) and 77 (38.3%) respectively. Only intermediate resistance to Penicillin G was seen as shown in table 2.

**Table 2:** Antimicrobial resistance pattern (%) for most common and vaccine containing serotypes found in pneumococcal carriage in Nigeria.

	Penicillin	TMP-SMX	Tetracycline	Cefotaxime	Erythromycin	Chloramphenicol
**Overall (201)**	62 (30.9)	187 (93.0)	169 (84.1)	1 (0.5)	0 (0.0)	17 (8.5)
**Top 10 Serotypes**						
**19F (33)**	13 (39.4)	30 (90.9)	28 (84.8)	1 (3.0)	0 (0.0)	2 (6.1)
**6A (31)**	8 (25.8)	31 (100)	29 (93.6)	0 (0.0)	0 (0.0)	3 (9.7)
**6B (26)**	4 (15.4)	24 (92.3)	25 (96.2)	0 (0.0)	0 (0.0)	5 (19.2)
**23F (16)**	4 (25.0)	16 (100.0)	13 (81.3)	0 (0.0)	0 (0.0)	0 (0.0)
**11 (16)**	2 (12.5)	13 (81.2)	6 (37.5)	0 (0.0)	0 (0.0)	2 (12.5)
**15B (7)**	1 (14.3)	7 (100.0)	7 (100.0)	0 (0.0)	0 (0.0)	1 (14.3)
**3 (7)**	6 (85.7)	7 (100.0)	7 (100.0)	0 (0.0)	0 (0.0)	0 (0.0)
**NT (9)**	6 (66.7)	9 (100.0)	8 (88.9)	0 (0.0)	0 (0.0)	1 (11.1)
**18C (3)**	0 (0.0)	3 (100.0)	3 (100.0)	0 (0.0)	0 (0.0)	0 (0.0)
**9V (4)**	0 (0.0)	4 (100.0)	4 (100.0)	0 (0.0)	0 (0.0)	0 (0.0)
**PCV-7 Serotypes (81)**	26 (29.9)	82 (94.3)	77 (88.5)	1 (1.2)	0 (0.0)	6 (6.9)
**PCV-10 Serotypes (88)**	26 (29.6)	83 (94.3)	78 (88.6)	1 (1.1)	0 (0.0)	6 (6.8)
**PCV-13 Serotypes (123)**	40 (32.5)	118 (95.9)	111 (90.2)	1 (0.8)	0 (0.0)	7 (5.7)

Antibiotic use was associated with age (p<0.001) and history of prior ingestion was highest in those aged <5 years (93 of 523, 17.8%) and aged >40 years (40 of 139, 29%); and least in the 5–14 years age category (6 of 125, 4.8%). Overall, there were no differences in prevalence of carriage by history of antibiotic use (52.3% vs. 54.0%, p = 0.7). When children (<18 years) were compared to adults, no differences in carriage by antibiotic use were seen as well.

### Assessment of risk factors for carriage

In multivariable analysis, pneumococcal carriage was adjusted for age, gender, overcrowding, predominant household cooking fuel, and total household residents aged <5 years. At the level of the individual, age and overcrowding were significantly associated with pneumococcal carriage. The odds of pneumococcal carriage in the <5 years age group was 7 times more than in those aged >40 years. They also had 2.5 times the odds of carriage found in the 5–14 years age group. As shown in table 3, the odds of pneumococcal carriage increased significantly with reducing age and this trend was significant (χ^2^ = 135.7, p<0.001). There was also a significant trend towards increased carriage with number of inhabitants (i.e. 4 or more occupants per room) in a household (χ^2^ = 4.3, p = 0.04). There was some evidence for reduced carriage with use of other cooking fuel (mainly charcoal and bottled gas) apart from kerosene but this was not statistically significant.

**Table 3:** Multivariable odds ratios determined by logistic regression for pneumococcal carriage and risk factors.

Risk factor	*OR (95% CI)	P- value	**OR (95% CI)	p-value
Age group (years)				
≥40	1.0		1.0	
15–39	1.1 (0.64–1.81)	<0.001	1.1 (0.65–1.91)	0.70
5–14	2.9 (1.69–5.03)	<0.001	2.9 (1.63–5.0)	<0.001
<5	7.1 (4.53–11.03)	<0.001	7.4 (4.60–11.85)	<0.001
Gender				
Female	1.0		1.0	
Male	1.1 (0.79–1.44)	0.65	1.1 (0.81–1.48)	0.57
Predominant cooking fuel				
Kerosene	1.0		1.0	
Other	0.4 (0.17–1.12)	0.08	0.5 (0.18–1.16)	0.10
Overcrowding index				
≤3	1.0		1.0	
≥4	1.3 (1.02–1.83)	0.03	1.3 (0.97–1.78)	0.07
Number of resident under fives				
0	1.0		1.0	
≥1	0.8 (0.60–1.01)	0.14	0.8 (0.61–1.11)	0.20

*OR-Odds Ratio from Logistic regression model.

**Adjusted for clustering.

On adjusting the data for clustering by location in a random effect logistic regression model, age remained the key risk factor for carriage at community level. The evidence for an association between overcrowding and pneumococcal carriage weakened when data was adjusted for clustering.

## Discussion

This study investigated pneumococcal carriage in a Nigerian population to determine carriage rates, prevalent serotypes, theoretical coverage of available pneumococcal conjugate vaccines and population biology of isolated pneumococci. Since recently acquired pneumococci in carriage are usually the primary source of serotypes that cause invasive disease, carriage of pneumococcus can provide information on prevalence of antimicrobial resistance and some insight into need for vaccines and potential impact of selected vaccine on IPD. [10], [31]

Nigeria is one of the countries in the west sub-Saharan Africa region that currently plans to introduce one of the available PCVs. This may likely be PCV-13 if the example of other African countries (Mali, Rwanda, Gambia and Sierra Leone) is followed. If introduced, the likely benefit of this vaccine is currently unknown and baseline data to inform expected changes or trends in pneumococcal carriage and IPD are unavailable. We hope this study will address some of these knowledge gaps. To our knowledge, this is the first investigation into the risk factors and population structure of carried pneumococcal in Nigeria.

The prevalence of pneumococcal carriage varies within country and setting ranging from <10% in some developed countries like Italy and France [32], [33], [34] to values >50% in indigenous children in developed countries and among populations in developing countries [13], [15], [35]. We found high levels of pneumococcal carriage in Nigeria as described in similar settings [12], [14], [36], [37]. The higher prevalence of pneumococcal carriage in children especially young infants compared to adults is also consistent with reports in the literature [14]. These findings are consistent with those from a smaller study of infants in South East, Nigeria [21].

The 42 pneumococci serotypes found in this study population provide the first detailed description of the repertoire of carried pneumococcal serotypes in Nigeria. The most common serotypes were 19F, 6A, 6B, 23F, 11 and 15B. Interestingly, these are among the most common serotypes reported in carriage studies in similar settings [14], [38]. Pneumococci serotypes carried by children <5 years are the major source of transmission to older children and adults within households and communities [28]. Vaccination with PCVs directly reduces carriage thus reducing transmission and there is a herd effect or benefit to communities among the unvaccinated populations [39]. Our findings here of high carriage rates especially in infants and children <2 years highlights the potential impact of PCVs on carriage and community transmission of pneumococcus if introduced in Nigeria.

Although data on serotypes implicated in IPD in Nigeria is limited, serotypes 19F, 4 and 5 were found in a recent report on surveillance of IPD [22] and serotypes 1, 2, 3 and 5 were reported in children <12 years over 30 years ago [19]. Since a third of isolates found in this carriage study belong to this group of serotypes previously reported to be associated with IPD in Nigeria, this implies IPD will be reduced significantly by introducing PCV-13 that contains these serotypes. However, we did not find any serotype 5 isolate and serotype 1 was only isolated in 2 subjects despite their frequent isolation in IPD [40], [41]. Since they are both are rarely seen or occur at very low frequencies in carriage, our findings are not surprising [14], [40]. Despite the low numbers of serotypes 1 and 5 found, 4 of the top 6 serotypes in this study are included in the list of the 7 serotypes most commonly found in IPD globally thus confirming the need to introduce PCV in this setting [6].

The serotype coverage for PCV-7 (44%) is limited in Nigeria and similar settings because of the few serotypes it contains. In particular, it does not contain serotypes 1 and 5 that have been implicated 25–30% of IPD found in low resource settings [41]. Although some countries are switching from PCV-7 to PCV-10, PCV-10 did not provide any significant added coverage over PCV-7 in our study population based on the prevalent serotypes we found in carriage. In contrast, the coverage for the PCV-13 was 62% in our study population as a whole and 70% in those <5 years. Our finding of highest serotype coverage for all licensed PCVs in very young children who bear most of the morbidity and mortality associated with IPD is evidence for the potential benefit of introducing PCVs into the Nigerian immunization schedule for children. The evidence from our data confirms PCV-13 as the best option. Taken together, our findings here in carriage i.e. 70% coverage of serotypes in children <5 years and the available information on prevalent serotypes in IPD suggest introduction of PCV-13 will significantly reduce morbidity and mortality associated with pneumococcus in the short and long term. The communal benefits of PCVs even of limited serotype coverage to all age groups in similar settings has been well described [42].

Although replacement by non-vaccine serotypes in both carriage and disease occurs following prolonged routine use of PCVs [16], this has not diminished the gains associated with introduction of PCV. Rather, this highlights the need for introduction of routine surveillance to confirm continued utility of PCV-13 once widely deployed in Nigeria.

MLST analysis shows not only a higher proportion of novel STs but also a highly diverse pneumococcal population structure in Nigeria which is consistent with other studies of carried pneumococcal population in similar settings like The Gambia and Ghana [43]. As most MLST studies thus far have focused on pneumococcus isolated in the USA and Europe although most pneumococcal disease occurs in the developing world [44] this result is not surprising; hence a considerable portion of the global diversity of pneumococcal is currently unexplored. Knowledge of prevailing STs, strains or serotypes to target in a vaccine is crucial in terms of future vaccine development in Africa because clonal groupings may differ substantially according to geographical regions and these varied clonal groupings may predominate at different periods. For example, the serotype 1-ST 217 identified in this study belongs to the ST217 clonal complex which predominates in Africa but rare in Europe or US. The ST 217 clonal complex is one of the most important lethal genotypes implicated in meningitis outbreaks in Ghana and Burkina Faso [45], [46].

We also found evidence of capsular switching in three STs (280, 310 and 5543). After the introduction of the pneumococcal conjugate vaccine, particular strains with genetic advantage may change their capsules from vaccine serotypes to non-vaccine serotype through capsular switching [47], [48]. It is important to enhance surveillance of pneumococcal disease in Africa prior to routine use of pneumococcal conjugate vaccine to allow the detection of capsular switching and monitoring of the long-term effectiveness of the conjugate vaccine in use.

The high frequency of resistance to commonly used antibiotics is a source of worry especially as TS, to which 93% of isolates were resistant, is one the WHO recommended first line antibiotic for treatment for respiratory tract infections in most developing countries [49]. In many developing countries with high burden of pneumococci in carriage and disease, high levels of resistance to TS and TC have also been reported [14], [41], [50], [51]. The pattern of resistance is not surprising considering these top the list of antibiotics purchased over the counter that are misused by caregivers in Nigeria and similar settings. However, it is unclear how in vitro resistance relates to clinical response to treatment with TS in pneumococcal disease [52]. Finding almost a third of isolates resistant to penicillin G (though entirely of the intermediate category) is also a cause for concern. This is in contrast to observations from The Gambia [14] and Kenya [53] where penicillin resistant pneumococci are not prevalent. This is probably associated with indiscriminate antibiotic use and poor regulatory oversight of drug supply including unlimited access to prescription antibiotics, which Nigeria has in common with other settings of penicillin resistance. Most of the antimicrobial resistant isolates were of vaccine-type (96%) and invasive serotypes suggesting use of PCVs will lower the prevalence of resistant pneumococci. All the isolates were fully susceptible to erythromycin but the emerging resistance to chloramphenicol especially with the observed resistance to first-line antimicrobials calls for intervention.

Age <5 years [35], concurrent acute respiratory tract infection [12], household smoke or cooking fuel [54], recent use of antibiotics [15], and overcrowding [32] are some of the risk factors that have been associated with pneumococcal carriage. The only significant individual risk factor for carriage found in this study was age and this association remained significant after adjusting for clustering of data. This is consistent with data from both developing and developed world settings [12], [35], [55]. That such risk factors as socioeconomic class and breast-feeding were not associated with carriage may reflect the lack of socioeconomic diversity in the study population. In addition, breastfeeding for prolonged periods is a cultural norm in this part of the world.

The absence of community age-distribution data during determination of sample size did not affect our results. The study did not have sufficient power for serotype specific sub analysis despite achieving the target sample size. Our risk factor analyses did not include day care or school attendance so the contribution if any of this exposure to pneumococcal carriage among children in this population is still unknown. However, this risk factor was not associated with carriage in other studies conducted in settings like ours [14], [15]. That people are in crowded environments in these settings regardless of whether they are at a day care, in school or at home is the likely explanation for this finding.

In conclusion, carriage of pneumococcus is very high in this Nigerian community indicating a high burden of pneumococcal disease. Young age is a major risk factor for carriage. Results from this study provide much needed data on the profile of prevalent pneumococcal serotypes and antimicrobial susceptibility patterns in a well-defined Nigerian population. Further research is required to quantify the burden of IPD, the serotypes involved, associated risk factors and outcomes especially as not all serotypes implicated in IPD are found in carriage. However, data from this study provides a starting point and baseline for future comparisons.

The consequence of findings from this study, and essential to the introduction of routine immunization against pneumococcus is the need for an extensive regional/nationwide surveillance of pneumococcal carriage, IPD and antimicrobial resistance that are crucial for vaccination impact assessment. The global vaccine preventable invasive bacterial disease (VP-IBD's) 3-tiered approach to sentinel surveillance should be the model implemented in Nigeria alongside introduction of PCV-13. This should start with tier-1 i.e. surveillance for suspected meningitis in children under five years across the country with rapid expansion to tier-2 level surveillance that includes cases of suspected pneumonia and septicaemia. Revisions to local antibiotic policy and prescription practice are required in light of the significant resistance seen to commonly used antibiotics.

## Supporting Information

Table S1 Pneumococcal serotypes and MLST distribution (new alleles are underlined).
(DOCX)

## References

[1] Black RE, Cousens S, Johnson HL, Lawn JE, Rudan I (2010). Global, regional, and national causes of child mortality in 2008: a systematic analysis.. Lancet.

[2] Rajaratnam JK, Marcus JR, Flaxman AD, Wang H, Levin-Rector A (2010). Neonatal, postneonatal, childhood, and under-5 mortality for 187 countries, 1970–2010: a systematic analysis of progress towards Millennium Development Goal 4.. Lancet.

[3] Rudan I, Boschi-Pinto C, Biloglav Z, Mulholland K, Campbell H (2008). Epidemiology and etiology of childhood pneumonia.. Bull World Health Organ.

[4] UNICEF and WHO (2006). Pneumonia: the forgotten killer of children.

[5] Dheda K, Pooran A, Pai M, Miller RF, Lesley K (2007). Interpretation of Mycobacterium tuberculosis antigen-specific IFN-gamma release assays (T-SPOT.TB) and factors that may modulate test results.. J Infect.

[6] Johnson HL, Deloria-Knoll M, Levine OS, Stoszek SK, Freimanis Hance L (2010). Systematic evaluation of serotypes causing invasive pneumococcal disease among children under five: the pneumococcal global serotype project.. PLoS Med.

[7] Bogaert D, De Groot R, Hermans PW (2004). Streptococcus pneumoniae colonisation: the key to pneumococcal disease.. Lancet Infect Dis.

[8] Block SL, Harrison CJ, Hedrick JA, Tyler RD, Smith RA (1995). Penicillin-resistant Streptococcus pneumoniae in acute otitis media: risk factors, susceptibility patterns and antimicrobial management.. Pediatr Infect Dis J.

[9] Boken DJ, Chartrand SA, Moland ES, Goering RV (1996). Colonization with penicillin-nonsusceptible Streptococcus pneumoniae in urban and rural child-care centers.. Pediatr Infect Dis J.

[10] Faden H, Duffy L, Wasielewski R, Wolf J, Krystofik D (1997). Relationship between nasopharyngeal colonization and the development of otitis media in children. Tonawanda/Williamsville Pediatrics.. J Infect Dis.

[11] Gray BM, Converse GM, Dillon HC (1980). Epidemiologic studies of Streptococcus pneumoniae in infants: acquisition, carriage, and infection during the first 24 months of life.. J Infect Dis.

[12] Russell FM, Carapetis JR, Ketaiwai S, Kunabuli V, Taoi M (2006). Pneumococcal nasopharyngeal carriage and patterns of penicillin resistance in young children in Fiji.. Ann Trop Paediatr.

[13] Soewignjo S, Gessner BD, Sutanto A, Steinhoff M, Prijanto M (2001). Streptococcus pneumoniae nasopharyngeal carriage prevalence, serotype distribution, and resistance patterns among children on Lombok Island, Indonesia.. Clin Infect Dis.

[14] Hill PC, Akisanya A, Sankareh K, Cheung YB, Saaka M (2006). Nasopharyngeal carriage of Streptococcus pneumoniae in Gambian villagers.. Clin Infect Dis.

[15] Rivera-Olivero IA, Bogaert D, Bello T, del Nogal B, Sluijter M (2007). Pneumococcal carriage among indigenous Warao children in Venezuela: serotypes, susceptibility patterns, and molecular epidemiology.. Clin Infect Dis.

[16] Farrell DJ, Klugman KP, Pichichero M (2007). Increased antimicrobial resistance among nonvaccine serotypes of Streptococcus pneumoniae in the pediatric population after the introduction of 7-valent pneumococcal vaccine in the United States.. Pediatr Infect Dis J.

[17] Akanbi MO, Ukoli CO, Erhabor GE, Akanbi FO, Gordon SB (2009). The burden of respiratory disease in Nigeria.. African Journal of Respiratory Medicine.

[18] (2007). World Health Statistics,Geneva.. http://www.who.int/whosis/whostat2007.pdf.

[19] Onyemelukwe GC, Greenwood BM (1982). Pneumococcal serotypes, epidemiological factors and vaccine strategy in Nigerian patients.. Journal of Infection.

[20] Silverman M, Stratton D, Diallo A, Egler LJ (1977). Diagnosis of acute bacterial pneumonia in Nigerian children. Value of needle aspiration of lung of countercurrent immunoelectrophoresis.. Arch Dis Child.

[21] Nwachukwu NC, Orji A (2008). Streptococcus pneumoniae carriage rates among infants in Owerri, Nigeria.. African Journal of Respiratory Medicine September.

[22] Falade AG, Lagunju IA, Bakare RA, Odekanmi AA, Adegbola RA (2009). Invasive pneumococcal disease in children aged <5 years admitted to 3 urban hospitals in Ibadan, Nigeria.. Clin Infect Dis.

[23] O'Brien K, Nohynek H, Group WPCW (2003). Report from a WHO Working Group: standard method for detecting upper respiratory carriage of Streptococcus pneumoniae.. Pediatr Infect Dis J.

[24] O'Brien KL, Bronsdon MA, Dagan R, Yagupsky P, Janco J (2001). Evaluation of a medium (STGG) for transport and optimal recovery of Streptococcus pneumoniae from nasopharyngeal secretions collected during field studies.. J Clin Microbiol.

[25] Antonio M, Dada-Adegbola H, Biney E, Awine T, O'Callaghan J (2008). Molecular epidemiology of pneumococci obtained from Gambian children aged 2–29 months with invasive pneumococcal disease during a trial of a 9-valent pneumococcal conjugate vaccine.. BMC Infect Dis.

[26] Melnick N, Thompson TA, Beall BW (2010). Serotype-specific typing antisera for pneumococcal serogroup 6 serotypes 6A, 6B, and 6C.. J Clin Microbiol.

[27] UK NEQAS United Kingdom National External Quality Assessment Service http://www.ukneqas.org.uk.

[28] Hill PC, Townend J, Antonio M, Akisanya B, Ebruke C (2010). Transmission of Streptococcus pneumoniae in rural Gambian villages: a longitudinal study.. Clin Infect Dis.

[29] Department of Infectious Disease Epidemiology IC, United Kingdom, eBURSTv3 http://eburst.mlst.net/.

[30] Oyedeji GA (1985). Socio-economic and Cultural Background of Hospitalised Children in Ilesha.. Nig J Paediatr.

[31] Syrjanen RK, Auranen KJ, Leino TM, Kilpi TM, Makela PH (2005). Pneumococcal acute otitis media in relation to pneumococcal nasopharyngeal carriage.. Pediatr Infect Dis J.

[32] Principi N, Marchisio P, Schito GC, Mannelli S (1999). Risk factors for carriage of respiratory pathogens in the nasopharynx of healthy children.. Pediatr Infect Dis J.

[33] Guillemot D, Carbon C, Balkau B, Geslin P, Lecoeur H (1998). Low dosage and long treatment duration of beta-lactam: risk factors for carriage of penicillin-resistant Streptococcus pneumoniae.. Jama.

[34] Marchisio P, Esposito S, Schito GC, Marchese A, Cavagna R (2002). Nasopharyngeal carriage of Streptococcus pneumoniae in healthy children: implications for the use of heptavalent pneumococcal conjugate vaccine.. Emerg Infect Dis.

[35] Ussery XT, Gessner BD, Lipman H, Elliott JA, Crain MJ (1996). Risk factors for nasopharyngeal carriage of resistant Streptococcus pneumoniae and detection of a multiply resistant clone among children living in the Yukon-Kuskokwim Delta region of Alaska.. Pediatr Infect Dis J.

[36] Jain A, Kumar P, Awasthi S (2005). High nasopharyngeal carriage of drug resistant Streptococcus pneumoniae and Haemophilus influenzae in North Indian schoolchildren.. Trop Med Int Health.

[37] Feikin DR, Davis M, Nwanyanwu OC, Kazembe PN, Barat LM (2003). Antibiotic resistance and serotype distribution of Streptococcus pneumoniae colonizing rural Malawian children.. Pediatr Infect Dis J.

[38] Hill PC, Cheung YB, Akisanya A, Sankareh K, Lahai G (2008). Nasopharyngeal carriage of Streptococcus pneumoniae in Gambian infants: a longitudinal study.. Clin Infect Dis.

[39] Lynch JP, Zhanel GG (2010). Streptococcus pneumoniae: epidemiology and risk factors, evolution of antimicrobial resistance, and impact of vaccines.. Curr Opin Pulm Med.

[40] Hausdorff WP, Feikin DR, Klugman KP (2005). Epidemiological differences among pneumococcal serotypes.. Lancet Infect Dis.

[41] Adegbola RA, Hill PC, Secka O, Ikumapayi UN, Lahai G (2006). Serotype and antimicrobial susceptibility patterns of isolates of Streptococcus pneumoniae causing invasive disease in The Gambia 1996–2003.. Trop Med Int Health.

[42] Roca A, Hill PC, Townend J, Egere U, Antonio M (2011). Effects of Community-Wide Vaccination with PCV-7 on Pneumococcal Nasopharyngeal Carriage in The Gambia: A Cluster-Randomized Trial.. PLoS Med.

[43] Donkor ES, Newman MJ, Oliver-Commey J, Bannerman E, Dayie NT (2010). Invasive disease and paediatric carriage of Streptococcus pneumoniae in Ghana.. Scand J Infect Dis.

[44] Gertz RE, McEllistrem MC, Boxrud DJ, Li Z, Sakota V (2003). Clonal distribution of invasive pneumococcal isolates from children and selected adults in the United States prior to 7-valent conjugate vaccine introduction.. J Clin Microbiol.

[45] Yaro S, Lourd M, Traore Y, Njanpop-Lafourcade BM, Sawadogo A (2006). Epidemiological and molecular characteristics of a highly lethal pneumococcal meningitis epidemic in Burkina Faso.. Clin Infect Dis.

[46] Leimkugel J, Adams Forgor A, Gagneux S, Pfluger V, Flierl C (2005). An outbreak of serotype 1 Streptococcus pneumoniae meningitis in northern Ghana with features that are characteristic of Neisseria meningitidis meningitis epidemics.. J Infect Dis.

[47] Kaplan SL, Mason EO, Wald ER, Schutze GE, Bradley JS (2004). Decrease of invasive pneumococcal infections in children among 8 children's hospitals in the United States after the introduction of the 7-valent pneumococcal conjugate vaccine.. Pediatrics.

[48] Huang SS, Platt R, Rifas-Shiman SL, Pelton SI, Goldmann D (2005). Post-PCV7 changes in colonizing pneumococcal serotypes in 16 Massachusetts communities, 2001 and 2004.. Pediatrics.

[49] World Health Organization (2008). Integrated Management of Childhood Illnesses.. http://whqlibdocwhoint/publications/2008/9789241597289_engpdf.

[50] Saha SK, Baqui AH, Darmstadt GL, Ruhulamin M, Hanif M (2003). Comparison of antibiotic resistance and serotype composition of carriage and invasive pneumococci among Bangladeshi children: implications for treatment policy and vaccine formulation.. J Clin Microbiol.

[51] Joloba ML, Bajaksouzian S, Palavecino E, Whalen C, Jacobs MR (2001). High prevalence of carriage of antibiotic-resistant Streptococcus pneumoniae in children in Kampala Uganda.. Int J Antimicrob Agents.

[52] Sazawal S, Black RE (2003). Effect of pneumonia case management on mortality in neonates, infants, and preschool children: a meta-analysis of community-based trials.. Lancet Infect Dis.

[53] Mudhune S, Wamae M (2009). Report on invasive disease and meningitis due to Haemophilus influenzae and Streptococcus pneumonia from the Network for Surveillance of Pneumococcal Disease in the East African Region.. Clin Infect Dis.

[54] Greenberg D, Givon-Lavi N, Broides A, Blancovich I, Peled N (2006). The contribution of smoking and exposure to tobacco smoke to Streptococcus pneumoniae and Haemophilus influenzae carriage in children and their mothers.. Clin Infect Dis.

[55] Dagan R, Givon-Lavi N, Zamir O, Sikuler-Cohen M, Guy L (2002). Reduction of nasopharyngeal carriage of Streptococcus pneumoniae after administration of a 9-valent pneumococcal conjugate vaccine to toddlers attending day care centers.. J Infect Dis.

