# Supplementary material for: Pre-Vaccination Nasopharyngeal Pneumococcal Carriage in a Nigerian Population: Epidemiology and Population Biology
Source: PLoS One. 2012 Jan 24;7(1):e30548. doi: 10.1371/journal.pone.0030548 (PMC3265474; doi:10.1371/journal.pone.0030548)
Supplement: Table S1 — Pneumococcal serotypes and MLST distribution (new alleles are underlined). (DOCX) [file pone.0030548.s001.docx]

**Table S1.**

|  |  | Allelic profile | | | | | | |  |
| --- | --- | --- | --- | --- | --- | --- | --- | --- | --- |
| Serotype | **ST** | ***aroE*** | ***gdh*** | ***gki*** | ***recP*** | ***spi*** | ***xpt*** | ***ddl*** | **No. of isolates** |
| 1 | 217 | 10 | 18 | 4 | 1 | 7 | 19 | 9 | 1 |
| 3 | 700 | 12 | 11 | 2 | 6 | 6 | 22 | 14 | 5 |
|  | 5534 | 7 | 43 | 4 | 154 | 6 | 3 | 8 | 2 |
| 4 | 2941 | 97 | 5 | 1 | 1 | 36 | 1 | 14 | 1 |
| 8 | 5535 | 10 | 13 | 34 | 1 | 15 | 111 | 31 | 1 |
| 11 | 3804 | 97 | 53 | 4 | 1 | 6 | 28 | 313 | 1 |
|  | 5536 | 7 | 2 | 4 | 4 | 15 | 173 | 18 | 1 |
|  | 5537 | 97 | 2 | 4 | 4 | 15 | 173 | 18 | 3 |
| 12 | 989 | 12 | 5 | 89 | 8 | 6 | 112 | 14 | 1 |
| 13 | 5538 | 13 | 9 | 4 | 18 | 15 | 1 | 9 | 1 |
| 14 | 63 | 2 | 5 | 36 | 12 | 17 | 21 | 14 | 3 |
|  | 3036 | 2 | 5 | 36 | 12 | 17 | 21 | 31 | 1 |
|  | 5561 | 176 | 5 | 36 | 12 | 17 | 21 | 14 | 1 |
|  | 5562 | 2 | 5 | 36 | 12 | 17 | 21 | 8 | 1 |
| 17 | 5539 | 12 | 19 | 2 | 8 | 6 | 22 | 14 | 1 |
|  | 5540 | 18 | 12 | 2 | 16 | 17 | 19 | 245 | 1 |
| 20 | 1794 | 10 | 16 | 54 | 1 | 15 | 1 | 31 | 1 |
| 21 | 5103 | 8 | 5 | 6 | 47 | 9 | 1 | 19 | 2 |
|  | 5563 | 2 | 5 | 6 | 12 | 17 | 21 | 14 | 1 |
| 38 | 5560 | 172 | 262 | 261 | 5 | 42 | 49 | 168 | 2 |
| 10F | 5564 | 2 | 8 | 4 | 16 | 6 | 1 | 309 | 1 |
| 15A | 5541 | 15 | 5 | 15 | 1 | 15 | 1 | 18 | 2 |
| 15B | 4033 | 118 | 8 | 4 | 12 | 17 | 19 | 6 | 1 |
|  | 5542 | 50 | 9 | 101 | 1 | 36 | 225 | 5 | 2 |
| 15B, 15C | 910 | 5 | 5 | 6 | 5 | 9 | 17 | 19 | 3 |
| 16F | 5565 | 177 | 134 | 4 | 18 | 1 | 83 | 18 | 1 |
| 18C | 1233 | 10 | 11 | 34 | 16 | 15 | 1 | 145 | 2 |
|  | 5574 | 10 | 11 | 34 | 16 | 244 | 1 | 145 | 1 |
| 19A | 230 | 12 | 19 | 2 | 17 | 6 | 22 | 14 | 1 |
|  | 847 | 7 | 11 | 4 | 1 | 6 | 112 | 14 | 1 |
|  | 5566 | 6 | 261 | 10 | 18 | 36 | 354 | 5 | 1 |
|  | 5567 | 12 | 19 | 2 | 17 | 7 | 22 | 5 | 1 |
| 19F | 1027 | 10 | 41 | 47 | 1 | 6 | 14 | 2 | 1 |
|  | 2773 | 50 | 5 | 123 | 1 | 36 | 83 | 74 | 1 |
|  | 4194 | 2 | 5 | 9 | 10 | 17 | 1 | 31 | 1 |
|  | 5568 | 6 | 5 | 2 | 5 | 17 | 1 | 17 | 1 |
|  | 5569 | 7 | 13 | 9 | 10 | 6 | 6 | 8 | 1 |
| 19F, 6A | 5543 | 1 | 134 | 101 | 18 | 130 | 1 | 6 | 3 |
| 23B | 172 | 7 | 13 | 8 | 6 | 25 | 6 | 8 | 1 |
|  | 5544 | 11 | 16 | 4 | 8 | 6 | 142 | 18 | 1 |
| 23F | 802 | 10 | 13 | 53 | 1 | 72 | 38 | 31 | 1 |
|  | 2520 | 7 | 16 | 8 | 8 | 6 | 142 | 235 | 2 |
|  | 5545 | 7 | 16 | 4 | 8 | 6 | 142 | 14 | 1 |
|  | 5570 | 7 | 5 | 8 | 8 | 15 | 355 | 235 | 1 |
| 35B | 5571 | 7 | 13 | 8 | 6 | 239 | 28 | 8 | 1 |
|  | 5572 | 8 | 134 | 70 | 1 | 13 | 346 | 8 | 1 |
| 38, NT | 310 | 1 | 43 | 41 | 18 | 13 | 49 | 6 | 3 |
| 6A | 361 | 7 | 13 | 8 | 6 | 6 | 6 | 8 | 1 |
|  | 2904 | 7 | 13 | 8 | 6 | 6 | 6 | 5 | 1 |
|  | 4011 | 7 | 13 | 8 | 6 | 6 | 1 | 8 | 1 |
|  | 5546 | 11 | 41 | 8 | 1 | 19 | 277 | 5 | 1 |
|  | 5547 | 7 | 19 | 8 | 10 | 6 | 6 | 8 | 1 |
|  | 5548 | 2 | 5 | 9 | 10 | 17 | 1 | 18 | 1 |
|  | 5549 | 7 | 13 | 8 | 10 | 6 | 6 | 8 | 1 |
|  | 5550 | 7 | 19 | 8 | 10 | 6 | 6 | 18 | 1 |
| 6B | 1348 | 7 | 5 | 4 | 12 | 6 | 20 | 8 | 1 |
|  | 3544 | 10 | 20 | 8 | 1 | 6 | 1 | 29 | 1 |
|  | 5551 | 7 | 260 | 260 | 6 | 17 | 60 | 14 | 1 |
|  | 5552 | 7 | 260 | 36 | 6 | 17 | 60 | 14 | 1 |
|  | 5553 | 2 | 37 | 9 | 12 | 17 | 21 | 59 | 1 |
|  | 5554 | 7 | 43 | 4 | 6 | 6 | 3 | 8 | 2 |
|  | 5555 | 12 | 28 | 1 | 2 | 6 | 26 | 9 | 1 |
|  | 5573 | 7 | 206 | 1 | 6 | 15 | 60 | 14 | 1 |
| 7C | 5556 | 1 | 8 | 146 | 15 | 25 | 231 | 14 | 1 |
|  | 5557 | 1 | 8 | 146 | 15 | 25 | 231 | 9 | 1 |
| 9V | 1871 | 1 | 16 | 4 | 10 | 7 | 1 | 31 | 1 |
|  | 4340 | 6 | 57 | 9 | 28 | 7 | 1 | 9 | 1 |
|  | 5692 | 7 | 77 | 1 | 2 | 15 | 60 | 406 | 1 |
| 9V, 6A | 280 | 15 | 17 | 4 | 16 | 6 | 1 | 17 | 3 |
| NT | 344 | 8 | 37 | 9 | 29 | 2 | 12 | 53 | 2 |
|  | 5558 | 7 | 77 | 2 | 1 | 130 | 4 | 31 | 1 |
|  | 5559 | 5 | 25 | 52 | 5 | 2 | 142 | 14 | 1 |
